# Is the elimination of ‘sleeping sickness’ affordable? Who will pay the price? Assessing the financial burden for the elimination of human African trypanosomiasis *Trypanosoma brucei gambiense* in sub-Saharan Africa

**DOI:** 10.1136/bmjgh-2018-001173

**Published:** 2019-04-14

**Authors:** C Simone Sutherland, Fabrizio Tediosi

**Affiliations:** 1 Institute of Pharmaceutical Medicine, Universitat Basel Medizinische Fakultat, Basel, Switzerland; 2 Department of Epidemiology and Public Health (EPH), Swiss Tropical and Public Health Institute, Basel, Switzerland; 3 Department of Public Health, University of Basel, Basel, Switzerland

**Keywords:** economics, sleeping sickness, neglected tropical diseases (NTDs), elimination, finance, budget, out-of-pocket, catastrophic health expenditures, financial protection, universal health coverage (UHC), sustainable development goals (SDG), *Trypanosoma brucei gambiense*, health policy decision-making

## Abstract

**Introduction:**

Programme to eliminate neglected tropical diseases (NTDs) have gained global recognition, and may allow for improvements to universal health coverage and poverty alleviation. It is hoped that elimination of human African trypanosomiasis (HAT) *Trypanosoma brucei gambiense (Tbg*) would assist in this goal, but the financial costs are still unknown. The objective of this analysis was to forecast the financial burden of direct costs of HAT *Tbg* to funders and society.

**Methods:**

In order to estimate the total costs to health services and individuals: (1) potential elimination programmes were defined; (2) the direct costs of programmes were calculated; (3) the per case out-of-pocket payments (OOPs) by programme and financial risk protection indicators were estimated. The total estimated costs for control and elimination programme were reported up till 2020 in international dollars. The mean results for both direct programme costs and OOPs were calculated and reported along with 95% CIs.

**Results:**

Across sub-Saharan Africa, HAT *Tbg* maintaining ‘Control’ would lead to a decline in cases and cost US$630.6 million. In comparison, the cost of ‘Elimination’ programme ranged from US$410.9 million to US$1.2 billion. Maintaining ‘Control’ would continue to cause impoverishment and financial hardship to households; while all ‘Elimination’ programme would lead to significant reductions in poverty.

**Conclusion:**

Overall, the total costs of either control or elimination programme would be near US$1 billion in the next decade. However, only elimination programme will reduce the number of cases and improve financial risk protection for households who are impacted by HAT *Tbg*.

**Summary Box**

**What is already known?**

- Financing sustainable health systems will increase Universal Health Coverage, but there are unknown costs related to elimination of neglected tropical diseases (NTDs); in particular, human African trypanosomiasis (HAT) *Trypanosoma brucei gambiense* (*Tbg*).
- The cost-effectiveness of strategies to eliminate HAT *Tbg* have proven to differ by foci, but how this translates into financial burden for funders and the communities at risk is still unknown.

**What are the new findings?**

- National programmes that combine varying cost-effective strategies and lead to elimination of HAT *Tbg* will cost millions of dollars in the coming decade, but will improve financial protection (alleviate poverty) by reducing the occurrence of catastrophic health expenditures to households impacted by HAT *Tbg*.
- Several countries burdened with sleeping sickness may not be able to afford optimal elimination programmes based on gross national income levels.

**What do the new findings imply?**

- Decision makers interested in elimination should not only consider the costs associated with the national programmes but also the societal perspective when prioritising programmes for the elimination of NTDs.
- Health financing and funding support will need to be addressed if elimination goals and sustainable development goals to eliminate poverty are to be achieved.

## Introduction

Over the last two decades, global health expenditures per capita have more than doubled, and continue to increase annually.^1^ In several countries a large proportion of the health expenditure is funded by patients’ out-of-pocket with severe consequences to the financial protection of the household. However, policy makers at all levels rarely take into account the potential consequences on a household’s economic conditions of health policies and interventions. In the last decade, neglected tropical diseases (NTDs) increasingly attracted the interest of global health investors and there is currently a global mandate to achieve disease elimination of several NTDs.^2^ In order to comprehensively define the economic implications for NTDs in particular for key stakeholders, an ‘Eradication’ or ‘elimination investment case’ (EIC) framework was developed.^3^ At present, this approach has been applied to *onchocerciasis* (‘river blindness’) and *lymphatic filariasis* (‘elephantiasis’), highlighting that investments in elimination or eradication of such diseases leads to economic, health and ethical benefits.^4–11^ Thus far, cost-effective elimination strategies for human African trypanosomiasis (HAT). *Trypanosoma brucei gambiense (Tbg*) have been identified,^12^ and the ethical considerations of each strategy have been formally assessed^13^; however, a summary of the direct costs required for national control and elimination programmes have yet to be ascertained.

HAT *Tbg*, also known as ‘sleeping sickness’ is caused by the presence of the *Tbg* parasite that is transmitted by the bite of a tsetse fly from human reservoirs.^14^ Symptoms of the disease in the early stages include fever, headaches, joint pain and itching^15^; however, second stage symptoms resemble more severe neurological elements as the parasite eventually crosses the blood brain barrier. Affected individuals in the second stage may display behaviour similar to that of a patient with mental illness leading to societal rejection, disdain and isolation even after the disease has been treated and the patient recovers.^16^ Although several sub-Saharan nations have tsetse inhabited areas with the potential of harbouring *Tbg,* currently there are only 13 endemic countries that still reported having HAT cases in 2013 according to the WHO Global Health Observatory.^17^ Patients infected with the disease are traditionally required to undergo a chemotherapy regimen at treatment centres that are accessible but often far from the villages of those most affected.^18^ This requires that patients be away from their families and absent from occupational obligations post-treatment resulting in financial consequences.^16^

Recent technological developments in treatments, surveillance approaches and diagnostics, along with feasible vector control interventions^19^ have shown that there are new alternatives to treat, identify and prevent HAT *Tbg* that are cost-effective. Decision makers now need to calculate the annual and future budget implications to sustain elimination strategies, as well as consider the economic implications for the communities involved. This study aims to estimate the potential costs associated with these two perspectives. First, to forecast the financial impact that elimination programmes may need to sustain elimination targets; second, to alert decision makers of the implications for financial risk protection to households affected by HAT *Tbg*.

## Methods

In order to estimate the direct costs and financial risk protection impacts, the current control and potential elimination programmes at a national level needed to be defined. Then for each programme it was necessary to estimate the associated direct costs, households’ out-of-pocket payments (OOPs) and financial risk protection indicators. Our approach considers both the perspective of the national and global funders who are interested in the direct costs associated with HAT *Tbg* elimination (including health services and vector control), and a households’ perspective (ie, OOPs). The mean results for both direct programme costs and household expenditures are calculated along with 95% CI to express the uncertainty surrounding the mean simulations. Costs are discounted at 3% and reported from 2013 and 2020 for a time horizon of 7 years.

### Defining control and potential elimination programmes

A priority setting exercise was undertaken in order to define optimal programmes according to cost-effectiveness and probability of elimination based on a previously published economic evaluation for strategies including new technologies in the elimination of *Tbg*.^12^ The micro-strategies originated from potential approaches to elimination foreseen with technologies that became available in 2013 and onwards described in Steinmann *et al*.^19^ The strategies ranged from adding new ‘tiny targets’ (small insecticide-impregnated screens) for vector control in addition to the current ‘screen and treat’ programmes that are readily available, to including a new oral tablet (oxaboroles) treatment that may eliminate the need for prolonged in-hospital treatment. These were further combined in Sutherland *et al* 12 and modelled as five mutually exclusive macro-strategies for elimination to determine cost-effective options per foci. These strategies for elimination were further combined into national control and elimination programmes, based on the following cost-effectiveness thresholds: US$200 per disability adjusted life year (DALY) averted, US$700 per DALY averted, and US$1500 per DALY averted. Each programme is briefly described within table 1, with additional details for the approach provided in appendix A1.

**Table 1:** Description of programmes for control and elimination

Programme	Cost-effectiveness threshold	Detailed description
**Control**	Reference	In alignment with the EIC methodology^3^ we use the ‘control’ as the counterfactual scenario which equates to recommendations by the WHO during 2013: total reliance on passive reporting in low transmission areas, biennial screening in moderate areas and annual screening in high transmission with the screening and treatment of CATT and pentamidine (stage1)/NECT (stage 2), respectively
**Elimination I**	~US$200 per DALY averted’	Involves the recommended surveillance levels for HAT *Tbg* by ‘Control’, but switching to new technologies for treatment (fexinidazole and oxaboroles) and diagnostics (rapid diagnostics with motorbike screening campaigns) in all areas but ***not*** implementing vector control strategies including ‘tiny targets’ (small insecticide-impregnated screens^38^)
**Elimination II**	~US$700 per DALY averted	Involves biennial surveillance in low risk transmission areas, currently recommended surveillance levels for HAT *Tbg* by WHO in moderate and high risk areas, switching to new technologies for treatment and diagnostics in all areas, ***and*** implementing vector control strategies including ‘tiny targets’ but **only** in high risk transmission areas
**Elimination III**	~US$1500 per DALY averted	Involves biennial surveillance in low risk transmission areas, currently recommended surveillance levels for HAT *Tbg* by WHO in moderate and high risk areas, switching to new technologies for treatment and diagnostics in all areas, ***and*** implementing vector control strategies including ‘tiny targets’ but **only** in moderate **and** high risk transmission areas

*WHO surveillance recommendations: low risk (no active surveillance, passive surveillance only), moderate—biennial surveillance, high—annual surveillance.

CATT, card agglutination trypanosomiasis test;DALY, disability adjusted life years;EIC, eradication investment case; HAT *Tbg*, human African trypanosomiasis *Trypanosoma brucei gambiense*;NECT, nifurtomix-eflornithine combination therapy.

#### Forecasting the financial impact of national programmes

A ‘bottom-up approach’ was used to estimate the total sub-Saharan costs. A dynamical transmission model developed by Stone and Chitnis^20^ has been used previously to estimate long-term costs and effects for control and elimination programmes for HAT *Tbg*.^12^ In our analysis, we exported the mean annual cost per person per foci (from 500 simulations) for each programme related to: surveillance (including diagnostics), treatment and vector control. These per person estimates were then projected to estimate country related costs based at risk populations areas^14^ and finally aggregated for a cumulative cost across the 13 endemic sub-Saharan countries.^21^

Cost functions related to the expenses incurred for surveillance, treatment, diagnostics and vector control programmes of *Tbg,* using the inputs listed in appendix A2, were developed and described previously in Sutherland *et al*.^12^ The dynamical transmission model was then run for various strategies by foci (eg, strategy A, strategy E, and so on) to generate an annual cost per person in an at risk transmission area.^12^ These per person estimates were taken directly from the model outputs and then multiplied to the at foci defined by WHO^14^ and calculated for current at risk populations of the endemic countries. The formula to represent these calculations can be described as follows in *Equation 1*:

Equation 1. Costs per foci

$$\sum_{t=1}^{30}\bar{C_{f}}={\left(c_{sur}+c_{tx}+\;c_{vc}\right)}_{f}\times n_{f}$$

where *t* represents the years and $\bar{C}$ are the mean costs over the 30 year time horizon. The costs for surveillance is *C_sur_,* treatment is *C_tx_* and vector control is *C_vc_*. All are represented as units per person in an at risk focus, hence *n* is the number of people at risk in a focus. A specific focus is represented by *f* for which there are three: low, moderate and high. Additional details for the strategy related costing are presented in Sutherland *et al*.^12^

Equation 2. Total costs per programme (including all foci)

$$\bar{TC}=\;{\bar{C}}_{low}+{\bar{C}}_{moderate}\;+{\bar{C}}_{high}$$

Hence, any given national programme (eg, Control, Elimination I, and so on) is the total cost of the low, moderate and high areas (refer to *Equation 2*).

#### Forecasting financial protection (financial protection analysis)

The estimated number of cases based on the four aforementioned programmes for control and elimination, were simulated across sub-Saharan Africa, again using the model published by Stone and Chitnis.^20^ These estimations are further described in appendix A3.1. It was assumed that each estimated *Tbg* case would represent a single household. A financial risk protection analysis (FPA) model was built in MS Excel 2010, and used Bayesian sampling techniques to estimate the household related data required for the FPA including: income, non-medical (NM) expenses (ie, food) and medical (M) expenses (ie, OOPs). Consumption (C), in our case the median income, of US$1360 per annum was derived from the average gross national income (GNI) of the 13 endemic nations impacted by *Tbg*, with costs of non-medical expenses (ie, food) derived from the literature.^22 23^ The total medical expenses paid by households was estimated from HAT studies found in the literature as well.^24^ A cost function per household OOP expenditure was then developed, taking into consideration that a family member or friend would attend the treatment clinic with the diagnosed individual, and was calculated as follows:

Equation 3. Out-of-pocket (OOP) household expenditures

$$\begin{array}{l} \sum_{t=1}^{30}\bar{OOP_{s_{i}}}=p_{i}\;\times \left[c_{fee\;}+\left(c_{accomodation\;}\;\times \left(tx_{days_{s_{i}}}+r_{days_{s_{i}}}\right)\right)\;+2\left(c_{transportation}\right)+2\left(c_{meals\;}\times \;\left(tx_{days_{s_{i}}}+r_{days_{s_{i}}}\right)\right)\right] \end{array}$$

where *p_i_* are the number of cases per stage, and *c* costs refer to meals (*c_meals_*), a return trip for transportation to the treatment facility (*c_transportation_*) and the per diem accommodation rates (*c_accomodation_*). The per diem treatment days defined as $tx_{{days}_{s_{i}}}$ are relative the treatment in the foreseen programme (ie, Control 2020, Elimination 2020, and so on), refer to appendix A3.2 for additional details.

Prices derived from the literature related to income and non-medical expenses, and OOPs related to HAT *Tbg*, were converted to international dollars (USD) using purchasing power parity listed in the World Economic Outlook database.^25^ Prices that were reported in Euros were converted to international dollars (USD) using the average exchange rates lists on the European Central Bank Statistical Data Warehouse. Once all costs were converted to USD, they were then inflated to 2013 dollars using average consumer price indices.^26^

The catastrophic health expenditures (CHEs) was calculated using the proportion of medical expenses related to HAT *Tbg* from a household’s total income, less a basic need (ie, food) as follows:

Equation 4. Catastrophic health expenditure (CHE)

$$\frac{M}{\left(C-NM\right)}$$

The proportions of CHE at 10% and 25% were defined by the sustainable development goal (SDG) 3.8.2 (30). For the purpose of this analysis, we assumed that only 36% of affected households would actually incur medical expenses.^27^ The rationale being that in many cases, households forgo payments that could potentially lead to catastrophic expenditures.^27^ We relax this assumption in the sensitivity analysis (SA). To visualise the impact that OOP had on a household relative to the poverty line (PL), a Pen Parades’ diagram, was generated. This image is often used to assist policy makers in reviewing the impact that OOP may have on subjecting households to poverty (impoverishing) or pushing families with an income below the PL, further into poverty (‘immiserising’). In addition, we also conducted a SA within the FPA to evaluate the impact on financial protection when there were: variations in the proportion of households that incur OOPs related to HAT *Tbg*, higher and lower definitions of the PL, and increases and decreases to the annual discount rate.

A normal distribution was applied to case estimates, while gamma distributions were applied to all cost inputs. A PL of US$1.9 per diem^28^ was used to account for the economic status of sub-Saharan Africa. It was assumed that the PL would be the same in 2013 and 2020. For each programme, 10 000 simulations were run to generate the mean reported outcomes and 95% CIs. To understand the potential impact of poverty with current and future technologies, the programme “Control 2013”^14^ was used as a comparative baseline measure to the alternative options. The outcomes of the FPA are reported across sub-Saharan African and by country income levels. Additional details for input parameters used in the FPA are provided in appendix A3.

#### Patient and public involvement statement

No patients or public participants were involved in this study.

## Results

### Financial impact

From 2013 until 2020, the cumulative costs of control and elimination programme of the strategies modelled are listed in table 2. The results demonstrate that maintaining the current control programme, without taking new technologies into consideration, will incur a total cost of US$630.6 million across sub-Saharan Africa. Introducing the first option of elimination programme (‘Elimination I’) leads to a total financial impact of US$410.9 million; while scaling up to ‘Elimination II’ would yield a total of US$988.0 million. Implementing ‘Elimination III’ across all sub-Saharan nations would lead to a total cost of US$1248.1 million. The net impact of each programme in comparison to ‘Control’ demonstrates that ‘Elimination I’ would actually lead to cost-savings, while net increases of US$357.4 million and US$617.5 million for ‘Elimination II’ and ‘Elimination III’, respectively.

**Table 2:** Financial impact, HAT *Tbg* programmes across sub-Saharan African

					Control 2020 (Counterfactual)		Elimination I 2020 ~US$200 per DALY averted		Elimination II 2020 ~US$700 per DALY averted		Elimination III 2020 ~US$1500 per DALY averted	
					**Total** (**USD million**)	**95%** CI (**USD million**)	**Total** (**USD million**)	**95%** CI (**USD million**)	**Total** (**USD million**)	**95%** CI (**USD million**)	**Total** (**USD million**)	**95%** CI (**USD million**)
Total, gross-financial impact*					630.6	630.3–631.0	410.9	410.7–411.1	988.0	987.6–988.5	1248.1	1247.2–1249.1
Total, net-financial impact†					NA	NA	−219.8	−219.6 to −219.9	357.4	357.3–357.5	617.5	616.9–618.1
**Country, income-level, gross-financial impact**												
	**GNI**		**Cases in 2013‡**	Population at risk* (×10^3^)								
Sub-Saharan Africa*			6228	54 958								
LIC	1006 or less		6105	45 325	546.97	546.65 to 547.28	353.05	352.77 to 333.14	765.29	764.92 to 765.34	998.19	997.45 to 999.04
LMIC	1006–3995		33	4369	23.15	23.13 to 23.15	17.82	17.80 to 17.82	116.90	116.84 to 116.96	129.68	129.61 to 129.76
UMIC§	3996–12 235		89	5209	60.51	60.47 to 60.55	40.08	40.06 to 40.11	105.85	105.79 to 105.91	120.21	120.12 to 120.28
**Country, gross-financial impact**												
CAR	330		59	555	6.94	6.93–6.94	4.5	4.45–4.46	8.66	8.66–8.66	11.00	10.99–11.01
DRC	380		5647	38 032	454.66	454.40–454.93	293.6	293.4–293.7	641.1	640.8–641.4	835.1	834.5–835.8
Guinea	470		78	1279	4.44	4.44–4.44	3.89	3.88–3.89	36.50	36.48–36.52	39.87	39.85–39.89
Uganda	680		9	2116	38.13	38.11–38.15	23.81	23.80–23.82	35.42	35.40–35.43	58.39	58.33–58.46
South Sudan	940		117	2397	34.00	33.98–34.02	21.59	21.58–21.61	33.08	33.06–33.11	41.25	41.21–41.29
Chad	980		195	946	8.80	8.79–8.80	5.66	5.66–5.66	10.53	10.52–10.53	12.58	12.57–12.59
Cameroon	1360		6	221	0.67	0.67–0.67	0.7	0.70–0.70	8.83	8.82–8.83	9.33	9.33–9.34
Cote d'Ivoire	1460		7	1300	5.46	5.46–5.46	4.52	4.51–4.52	37.43	37.41–37.45	41.58	41.55–41.60
Republic of the Congo	2710		20	2380	17.01	17.00–17.02	11.53	11.52–11.53	40.16	40.14–40.18	48.29	48.26–48.32
Nigeria	2970		0	468	0.01	0.01–0.01	1.07	1.07–1.07	30.48	30.47–30.50	30.48	30.47–30.50
Angola	4850		69	4300	59.15	59.11–59.19	38.85	38.83–38.88	93.86	93.81–93.91	107.36	107.28–107.43
Gabon	9450		17	878	0.61	0.61–0.61	0.76	0.76–0.76	11.30	11.29-11-31	11.68	11.67–11.68
Equatorial Guinea	12 640		3	31	0.75	0.75–0.75	0.47	0.47–0.47	0.69	0.69–0.69	1.17	1.17–1.17

Blue indicatescountries that can afford the Elimination programme using GNI as cost-effectiveness threshold.

*Includes countries with endemic cases from 2000 to 2014 reported inFranco *et al*.^37^

†Net budget impact compared to ‘Control’.

‡Includes 13 endemic countries in 2013, excluding Ghana.

§Includes UMIC and one HIC (Equatorial Guinea GNI greater than USD 12 236).

CAR, Central African Republic;DALY, disability adjusted life years; DRC, Democratic Republic of Congo; GNI, gross national income; HAT *Tbg*, human African trypanosomiasis *Trypanosoma brucei gambiense*; HIC, high-income country; LMIC, low-income country and middle-income country; NA, not applicable; UMIC, upper-middle income country; USD, US dollar.

If one evaluates the proportion of programme costs by GNI country levels, 80%–87% of financial burden for HAT *Tbg* will allotted to low-income countries (LICs) (refer table 2). In particular, the Democratic Republic of the Congo (DRC), which has the second lowest income of all the endemic sub-Saharan countries, but the highest number of cases, would be responsible for 65%–72% depending on the programme selected. Further more, if the GNI of a country is used as a proxy to determine a cost-effectiveness threshold, all countries could scale up to ‘Elimination I’, while 77% could consider ‘Elimination II’. Less than half (46%) of the endemic countries would be able to consider ‘Elimination III’ as good value for money using GNI as a threshold.

**Figure 1:**
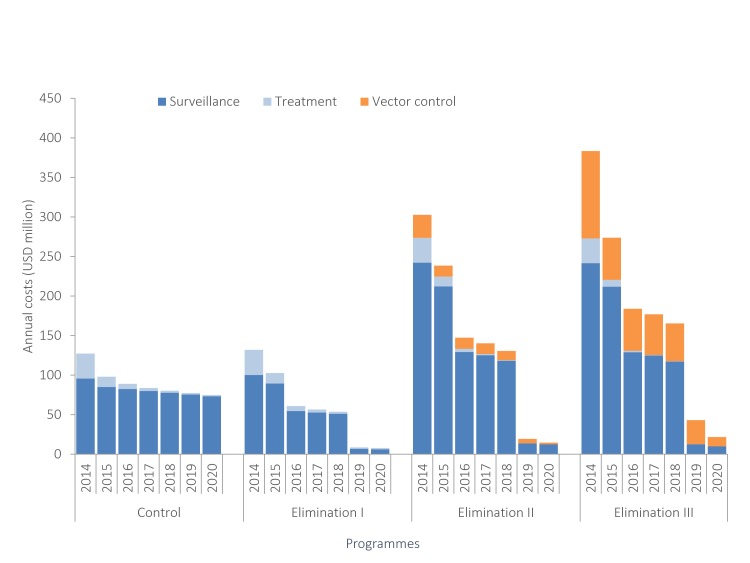
The year 2014 includes costs from 2013 and 2014 combined; USD, US dollar.

For each programme, as depicted in figure 1, the majority of costs for control and elimination programmes are driven by screening and diagnostic costs that come from passive surveillance, and/or active screening campaigns (68%–90% of total costs). The ongoing costs for control also begin to plateau after several years; while, although the costs of elimination programmes are high in the earlier years, they decline to 3%–5% of the overall costs in later years. The additional cost of vector control to ‘Elimination II’ and ‘Elimination III’ programmes contribute to 9% and 29% of the total programme costs, respectively.

### Financial risk protection

**Figure 2:**
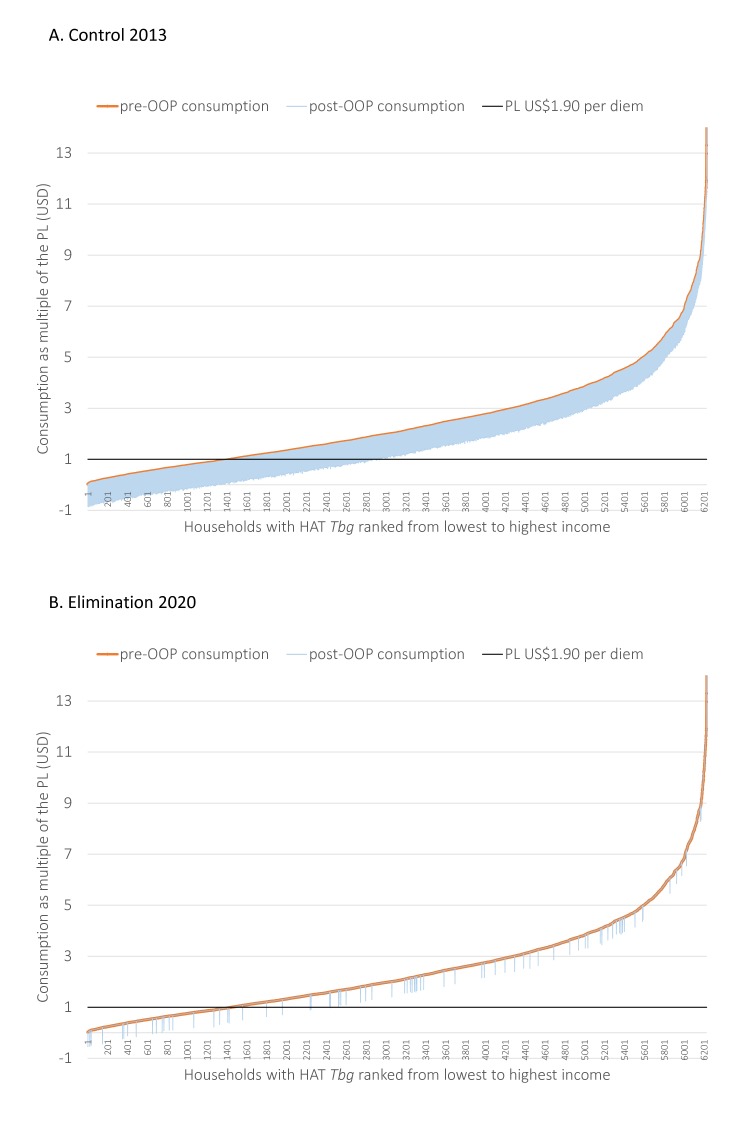
The Pen’s Parade diagramdepicts each household and their respective incomes pre-consumption (prior toincurring non-medical (NM) medical expenses (M) related to HAT *Tbg* and post-consumption (householdincome after incurring non-medical (NM) needs and medical (M) HAT *Tbg* expenses); HAT Tbg, human African trypanosomias Trypanosoma brucei gambiense; OOP, out-of-pocket payment; PL, poverty line; USD, US dollar.

The total number of expect cases and impacts related to poverty for all programmes completed in the financial protection analysis (FPA) are summarised in table 3. Overall, in comparison to the baseline year 2013, maintaining a similar ‘Control’ programme till 2020 will not have an impact on poverty indices. Although the number of cases were forecasted to decline, the OOP health expenditures related to attending treatment centres for HAT treatment will still lead to impoverishment (22%), immiserisation (31%) and CHEs at both 10% and 25% of HAT *Tbg* households. Scaling up to ‘Elimination’ programmes, that result in medications that can be taken at home or within local villages, will reduce the risk of impoverishment in the future by at least 5% for all elimination programmes in comparison to control and reduce the number of households facing CHEs to less than 1% (a reduction of 63%–30% depending on the thresholds selected). In addition, ‘Elimination’ programme lead to the fewest number of forecasted cases in the future. This dual impact is on households with *Tbg* is shown in the series of Pen’s Parade diagrams illustrated in figure 2.

**Table 3:** Financial protection: poverty impact of out-of-pocket payments related to HAT *Tbg* households in sub-Saharan Africa for control and elimination programmes

	Control 2013 (95% CI)	Control 2020 (95% CI)	Elimination I 2020 (95% CI)	Elimination II 2020 (95% CI)	Elimination III 2020 (95% CI)
**Total cases (N**)	6228*	2319	1768	76	56
	***Proportion (%) of HAT Tbg households***				
**Sub-Saharan Africa**					
Impoverishing	22.00% (21.96 to 22.03)	21.99% (21.94 to 22.04)	15.64% (15.59 to 15.70)	15.48% (15.23 to 15.73)	15.41% (15.11 to 15.71)
Immiserising	30.95% (30.92 to 30.99)	30.95% (30.90 to 31.01)	30.95% (30.88 to 31.01)	30.94% (30.61 to 31.27)	30.91% (30.53 to 31.30)
Catastrophic (CHE) at 10%	62.89% (62.85 to 62.93)	62.93% (62.87 to 62.99)	1.25% (1.23 to 1.27)	1.27% (1.20 to 1.35)	1.23% (1.14 to 1.32)
*Difference in % from control 2013*	NA	0.04% (0.02 to 0.06)	−61.64% (−61.61 to −61.66)	−61.61% (−61.65 to −61.,57)	−61.66% (−61.71 to −61.61)
Catastrophic (CHE) at 25%	30.95%	30.96% (30.90 to 31.02)	0.49% (0.48 to 0.50)	0.51% (0.46 to 0.56)	0.48% (0.43 to 0.54)
*Difference in % from control 2013*	NA	0.01% (−0.01 to 0.04)	−30.46% (−30.44 to −30.49)	−30.44% (−30 to 45 to −30.42)	−30.46% (−30.49 to −30.44)
**LIC**					
Impoverishing	15.04% (15.02 to 15.07)	15.09% (15.04 to 15.13)	14.98% (14.92 to 15.03)	15.23% (14.98 to 15.48)	15.02% (14.71 to 15.32)
Immiserising	84.95% (84.93 to 84.98)	84.91% (84.87 to 84.96)	84.95% (84.89 to 85.00)	84.72% (84.47 to 84.97)	84.89% (84.59 to 85.19)
Catastrophic (CHE) at 10%	99.44% (99.43 to 99.45)	99.43% (99.42 to 99.44)	0.86% (0.84 to 0.87)	0.82% (0.76 to 0.88)	0.91% (0.83 to 0.99)
*Difference in % from control 2013*	NA	−0.01% (−0.02 to −0.01)	−98.58% (−98.59 to −98.57)	−98.62% (−98.68 to −98.56)	−98.53% (−98.61 to −98.46)
Catastrophic (CHE) at 25%	98.80% (98.80 to 98.81)	98.78% (98.77 to 98.80)	0.24% (0.24 to 0.25)	0.25% (0.21 to 0.28)	0.26% (0.22 to 0.30)
*Difference in % from control 2013*	NA	−0.02% (−0.03 to −0.01)	−98.56% (−98.56 to −98.56)	−98.56% (−98.58 to −98.53)	−98.54% (−98.58 to −98.51)
**LMIC**					
Impoverishing	0.49% (0.49 to 0.50)	0.49% (0.48 to 0.50)	0.08% (0.08 zo 0.09)	0.09% (0.07 to 0.11)	0.10% (0.07 to 0.13)
Immiserising	0.00% (NA)	0.00% (NA)	0.00% (NA)	0.00% (NA)	0.00% (NA)
Catastrophic (CHE) at 10%	29.07% (29.04 to 29.11)	29.07% (29.01 to 29.13)	0.00% (NA)	0.00% (NA)	0.00% (NA)
*Difference in % from control 2013*	NA	0.00% (−0.03 to 0.02)	−29.07% (−29.04 to −29.11)	−29.07% (−29.04 to −29.11)	−29.07% (−29.04 to −29.11)
Catastrophic (CHE) at 25%	0.03% (0.03 to 0.03)	0.03% (0.03 to 0.03)	0.00% (NA)	0.00% (NA)	0.00% (NA)
*Difference in % from control 2013*	NA	0.00% (NA)	−0.03% (−0.03% to −0.03%)	−0.03% (−0.03% to −0.03%)	−0.03% (−0.03% to −0.03%)
**UMIC†**					
Impoverishing	0.00% (NA)	0.00% (NA)	0.00% (NA)	0.00% (NA)	0.00% (NA)
Immiserising	0.00% (NA)	0.00% (NA)	0.00% (NA)	0.00% (NA)	0.00% (NA)
Catastrophic (CHE) at 10%	0.00% (NA)	0.00% (NA)	0.00% (NA)	0.00% (NA)	0.00% (NA)
*Difference in % from control 2013*	NA	0.00% (NA)	0.00% (NA)	0.00% (NA)	0.00% (NA)
Catastrophic (CHE) at 25%	0.00% (NA)	0.00% (NA)	0.00% (NA)	0.00% (NA)	0.00% (NA)
*Difference in % from control 2013*	NA	0.00% (NA)	0.00% (NA)	0.00% (NA)	0.00% (NA)

N denotes number of population.

*Estimated cases from WHO (Franco *et al* 37).

†Includes UMIC and one HIC (Equatorial Guinea GNI greater than US$12 236).

CHE, catastrophic health expenditure; GNI, gross national income; HAT *Tbg*, human African trypanosomiasis *Trypanosoma brucei gambiense*; HIC, high-income country; LMIC, low-income country and middle-income country; NA, not applicable; UMIC, upper-middle income country.

Additional FPAs were evaluated by country income levels (refer table 3). The results again demonstrate that the (LICs) would be the most vulnerable to OOPs with 99.4% and 98.8% of the households experiencing CHEs at 10% and 25%, respectively if ‘Control’ is maintained. In addition, although at first glance it appears that there are fewer being impoverished, this is only due to the fact that the majority of the households in LICs will risk immiserisation (approximately 85%) for healthcare payments related to HAT, even with ‘Elimination’ programmes. Low-income countries and middle-income countries (LMICs) have smaller populations of households at risk of impoverishment (0.49%) and CHEs when ‘Control’ is in place, however; introducing ‘Elimination’ programme could also eliminate CHEs and reduce the proportion of those impoverished to less than 0.5%. There is not foreseen risk to poverty related to HAT *Tbg* expenditures in upper-middle income countries (UMICs).

The results from the univariate SA of the FPA across sub-Saharan Africa did not deviate from the base case analysis when changes in the discount rate were completed, or the PL was lowered to US$1.25. In additional, even when 60%–75% of households paid the OOPs related to HAT *Tbg,* the FPA results remained similar. However, if only 36% of households incur OOPs when a family member is diagnosed with HAT, the impoverishing and immiserising proportions would be similar across all programmes, with fewer people experiencing CHEs. Nonetheless, the ‘Elimination’ programmes would still be the only programmes that could fully alleviate households from experiencing CHEs. The results of the SA are available in appendix A3.

## Discussion

Across sub-Saharan Africa, HAT *Tbg* control and elimination programmes will require substantial funding and fiscal commitments over the coming years. A ‘Control’ programme will lead to fewer cases in the coming years, but it will lead to delays in reaching elimination targets and it will still cost millions of dollars to funders. In addition, a ‘Control’ programme will have no impact on reducing OOPs and poverty indices encouraged by the SDGs. Moving to ‘Elimination’ is an attractive option, but the ‘Elimination’ programmes that are most likely to reduce cases and alleviate OOPs related to HAT *Tbg* (Elimination II and III), are relatively expensive for the countries that share the greatest burden of at risk communities. The majority of the incremental funding required will need to be allocated to in-country surveillance and diagnostics annually. This alerts a cause for concern for stakeholders seeking elimination, as traditionally only the treatments for HAT *Tbg* have been donated with the majority of healthcare costs reliant on non-profit organizations (NGOs) and the fragile national health system. In addition, in order for these programmes to reach the at risk population, substantial logistical and technical capacity would be required^29 30^ which is not fully reflected in the simulated costs of the programmes.

The results of this two-part analysis demonstrate that there is a need for the global health community to prepare substantial funds for elimination, not only for treatments and preventative measures (ie, vector control) but also to the health system itself. In addition, it has demonstrated that the strive to elimination programmes may alleviate the households’ risk of impoverishment and thus contributing to reach the millennial SDGs.

The results of the analysis on financial burden highlight that ‘Elimination’ programmes are the most favourable in achieving global goals. However, decision makers will have to assess the fiscal space needed especially since availability of new HAT treatments has historically had delays in Pan-African uptake in the market. There may be a risk that LICs will opt for the less aggressive ‘Elimination I’ programme that would be cheaper. However, this will lead to a slower decline in cases compared with other elimination options and it could jeopardise the long-term goal of eliminating HAT. In addition, although the costs for OOPs expenses and direct programme costs have been converted to international dollars for ease of comparison, the true heterogeneity of costs across sub-Saharan still need to be captured which will require cost data collection at the national level at the very least.

In addition, HAT is not the only NTD ear-marked for elimination/eradication, and so it must be kept in mind that there is still competition for funds even though the costs of elimination are known.^9^ This may pose a challenge for HAT during prioritisation of funding for NTDs. For example, the onchocerciasis, eradication and elimination programmes are cost-saving relative to control and the per person at risk cost for HAT remains a relatively costly disease with programme ranging from US$3 to US$10 per person annually compared with ranges of US$1.5–US$3.9 per treatment in the onchocerciasis programme.^9^

HAT *Tbg* is already known to be a disease that affects the poor, but the current results demonstrate that households who are already near the PL, may fall: closer to the PL, under the PL (impoverished), or further into poverty (immiserised). However, the results of this analysis are still conservative as, recent evidence shows that there are often additional expenses prior to the diagnosis of HAT as households seek alternative care and guidance when family members are ill.^31^

Although the methodology for poverty assessments using household surveys has been established,^28 32^ the approach of modelling prospectively to understand its impact on future technologies is novel. The FPA presented here is also not based on household surveys and instead relies on secondary data and modelling to generate conclusions. However, it demonstrates the potential to provide useful information in a decision-making context for new technologies. In addition, the FPA does not address other societal costs, such as the loss of wages, which has been done within an eradication investment case for other neglected diseases.^5 9^ These costs have recently been estimated along with other NTDs for elimination by Lenk *et al*,^33^ and demonstrate that the contribution of productivity loss to HAT is substantial.^33^

There are also economic gains from elimination that could be considered in future EIC assessments. For instance, tsetse free areas may increase access to water areas without menace and even land use opportunities. Future research in prospective FPA could consider if and how additional income to at risk communities (ie, households) may result from elimination and assist to offset OOPs related to CHEs.

Although this analysis highlights the financial benefits of elimination programmes, at the time of its inception, it was assumed that novel interventions would arrive on the market as scheduled. The efficacy of fexinidazole has been proven^34^ and, while the European Medicines Agency (EMA)’s Committee for Medicinal Products for Human Use has adopted a positive scientific opinion in November 2018, the timeframe for its actual deployment is uncertain. In addition, previous modelling^12^ estimated the arrival of a novel one time treatment in 2018, while it is currently estimated to be submitted to the EMA in 2021.^35^ In the interim, subsidies for travel, accommodation and food could be considered, with the current national sleeping sickness control programmes (NSSCPs) to encourage patients to attend without the risk of incurring in financial hardship. Nonetheless, the scale up of elimination activities has begun in at least nine endemic nations,^36^ and there is a continual decline in cases supporting the model’s estimations.

Our results also assume 80% coverage is maintained for surveillance programmes. This coverage rate shows promise in leading to elimination but may be hard to achieve. Although 30% of the high transmission areas are covered, on average, only 2% of the continent is experiencing some level of screening. Furthermore, there are several countries that have not established formal NSSCPs (ie, Gambia, Guinea-Bissau, Liberia), although foci risk areas exist.^37^ Hence, the scale up to elimination will be substantial not only in funds, but for logistics on the ground. Scaling up coverage may reveal also that there are more cases than once thought, and adjustments to the elimination forecast and budgets will have to be made. Long-term commitments to funding postelimination will be needed as the possibility of asymptomatic cases and unknown reservoirs comes to the forefront. An example of this was seen recently in Ghana where one case was found in 2013 after 12 years of no cases being reported.

## Conclusion

Overall, the results demonstrate that an ‘Elimination II’ foci–specific programme that deploys: targets in high-risk areas with annual surveillance, adopts new technologies in all areas and implements biannual surveillance in moderate and low transmission zones, remains within cost-effectiveness thresholds for some low and lower-middle income countries. It could also achieve elimination goals for 2020 in the long-run and leads to financial protection for families impacted by HAT *Tbg*. Global stakeholders and funders should ensure that low-income countries whose NSSCPs are unable to secure funds nationally for elimination should be supported as they share a disproportionate load of the disease burden. The elimination of HAT *Tbg* does have a high cost, but with continued efforts and support from global stakeholders, it is hoped that those already at risk of poverty will not be the ones to pay for it.

10.1136/bmjgh-2018-001173.supp1 Supplementary data
