# Supplementary material for: Is the elimination of ‘sleeping sickness’ affordable? Who will pay the price? Assessing the financial burden for the elimination of human African trypanosomiasis Trypanosoma brucei gambiense in sub-Saharan Africa
Source: BMJ Glob Health. 2019 Apr 14;4(2):e001173. doi: 10.1136/bmjgh-2018-001173 (PMC6509604; doi:10.1136/bmjgh-2018-001173)
Supplement: Supplementary data [file bmjgh-2018-001173supp001.pdf]

APPENDIX

Contents

A.1 Defining national programs using a “rationale choice” approach 2

A.2 Financial forecast, health care expenditures 3

National ‘screen & treat’ programs and vector control program costs 3

A.3 Financial protection analysis 4

A.3.1 Case estimations 4

A.3.2 Out-of-pocket (OOP) Household health expenditures related to T.b. gambiense 6

References 10

Figure 1. Number of cases (households) actual (WHO surveillance) and forecasted (Stone & Chitnis 2015, Sutherland et al 2017) till 2020 5

Table 1. Cost-effectiveness of strategies by foci (Sutherland et al, 2017) 2

Table 2. Cost-effectiveness of strategies by foci (Sutherland et al, 2017), including strategy D+ from sensitivity analyses 2

Table 3. Potential national programs for control and elimination using variations in cost-effective strategies per foci 3

Table 4. Input parameters, costs and resource use related to control and elimination programs (funders) 3

Table 5. Number of cases (households) forecasted (Stone & Chitnis 2015, Sutherland et al 2017) till 2020 for all programs: Control, Elimination I, Elimination II, and Elimination III (based on 500 simulations) 5

Table 6. Costs and resource use related to OOP household expenditures 6

Table 7. Summary of inputs for the Financial Protection Analysis (FPA) 7

Table 8. Sensitivity and scenario analyses results for FPA, sub-Saharan Africa 8

A.1 Defining national programs using a "rationale choice" approach

The programs for Control, Elimination I, Elimination II and Elimination III described in the main manuscripts were developed using a "rationale choice approach". During priority setting for new technologies within a given budget, decision makers may use a "rational choice" approach that includes choosing the most cost-effective interventions. In this case, it was assumed that the incremental cost-effectiveness ratios (ICERs) would guide which strategies per foci should be selected for a national elimination program based on cost-effective as per Sutherland et al 2017. The results listed in *Table 1* indicate the cost-effectiveness of program per foci reported in Sutherland et al 2017.[1] These results show that within high and moderate risk transmission areas, strategy E (implementation of new technologies and tiny targets for vector control) would be cost-effective at $386 and $1509 per DALY averted respectively. In low transmission areas, incorporating new technologies without novel vector control methods would be cost-effective at $160 per DALY averted, while adding tiny targets to the new technologies would be cost-effective at $1812 per DALY averted.

Table 1. Cost-effectiveness of strategies by foci (Sutherland et al, 2017)

**Strategies (High risk transmission)**	**Cost**	**DALYs**	**ICER ($ per DALY averted)**
Strategy D, new technologies 2016 and 2019	$45	0.22	
Strategy C, new technologies 2016	$47	0.25	Dominated by Strategy D
Strategy E, new technologies 2016 and 2019 plus tiny targets	$61	0.18	$386
Strategy B, control with tiny targets	$82	0.20	Dominated by Strategy E
Strategy A, control	$115	0.34	Dominated by Strategy E
**Strategies (Moderate risk transmission)**	**Cost**	**DALYs**	**ICER**
Strategy D, new technologies 2016 and 2019	$20	0.03	-
Strategy C, new technologies 2016	$20	0.03	Dominated by Strategy D
Strategy E, new technologies 2016 and 2019 plus tiny targets	$38	0.02	$1509
Strategy B, control with tiny targets	$48	0.02	Dominated by Strategy E
Strategy A, control	$55	0.04	Dominated by Strategy E
**Strategies (Low risk transmission)**	**Cost**	**DALYs**	**ICER**
Strategy C, new technologies 2016	$3	0.04	
Strategy A, control	$3	0.04	Dominated by Strategy C
Strategy D, new technologies 2016 and 2019	$3	0.03	$160
Strategy E, new technologies 2016 and 2019 plus tiny targets	$42	0.01	1812
Strategy B, control with tiny targets	$45	0.01	Dominated by Strategy E

DALY= disability adjusted life year, ICER= incremental cost-effectiveness ratio

However, the sensitivity analysis (SA) demonstrated that increased surveillance could be cost-effective (Refer to *Table 2*). When it was compared to the current interventions it was the dominant choice (cost less, more effects); both strategy E and B were dominated by it. Hence, strategy D plus bi-annual surveillance could be considered a cost-effective strategy for decision makers with a cost-effectiveness threshold near $650 per DALY averted or greater.

Table 2. Cost-effectiveness of strategies by foci (Sutherland et al, 2017), including strategy D+ from sensitivity analyses

**Strategies (Low risk transmission)**	**Cost**	**DALYs**	**ICER**
Strategy C, new technologies 2016	$3	0.04	
Strategy A, control	$3	0.04	Dominated by Strategy C
Strategy D, new technologies 2016 and 2019	$3	0.03	$160

Strategy D+, new technologies 2016 and 2019 +bi-annual surveillance	*$20*	*0.004*	*$654*
Strategy E, new technologies 2016 and 2019 plus tiny targets	$42	0.01	Dominated by Strategy D+
Strategy B, control with tiny targets	$45	0.01	Dominated by Strategy D+

Therefore it was decided that three options (strategies A, D or D+) could potentially be available in low transmission areas depending on cost-effectiveness thresholds (near or less than $1500 per DALY averted) and feasibility of running additional surveillance programs. This cost-effectiveness threshold was considered, as the median of the gross national incomes (GNIs) from the impacted nations was near $1410. In cases where there was no ICER since the strategy was the comparator, the strategy with the lowest cost was considered. Refer to *Table 3*.

Table 3. Potential national programs for control and elimination using variations in cost-effective strategies per foci

Program plan	Cost-effectiveness threshold	Strategy per foci (ICER)		
		Low	Moderate	High
**Control**	Reference	A	A	A
**Elimination I**	~$300 per DALY averted	D ($160)	D (lowest cost)	D (lowest cost)
**Elimination II**	~$700 per DALY averted	D+ ($654)	D (lowest cost)	**E ($386)**
**Elimination III**	~$1500 per DALY averted	D+ ($654)	**E ($1509)**	**E ($386)**

A.2 Financial forecast, health care expenditures

National 'screen & treat' programs and vector control program costs

Table 4. Input parameters, costs and resource use related to control and elimination programs (funders)

	**Mean estimate Cost $US, (SD)**	**Sources**
**Financial costs**		
Surveillance	*Cost per person in at risk transmission area*	
**Annual surveillance**	0.42	Lutumba 2007
**Mobile team start-up**	1.55	Lutumba 2007
**Mobile teams annual maintenance**	0.21	Lutumba 2007
**Motorbike team start-up**	0.89	Lutumba 2007
**Motorbike team annual maintenance**	0.42	Lutumba 2007
**Passive surveillance**	1.35	Lutumba 2007
Diagnostics		
**CATT algorithm**	8.19	Lutumba 2005,WHO Technical report 1998, Lutumba 2006
**RDT 1 algorithm**	6.17	Lutumba 2006,Ndung'u 2015,FIND
**RDT 2 algorithm**	3.86	FIND
Treatment*	*Cost per person in at risk transmission area*	
**Pentamidine**	367.56	Shaw 2001, Politi 1995
**NECT**	816.46	Simarro 2011 and 2012
**fexinidazole**	55.23	DNDi

**oxaborole**	2.00	DNDi
Vector control program	*Cost per km in at risk transmission area*	
**Tiny targets start-up**	13.8	Shaw 2015
**Tiny target maintenance**	13.8	Shaw 2015

SD = standard deviation,*(per diem, Including hospitalization)

A.3 Financial protection analysis

A.3.1 Case estimations

The modelling simulates three foci areas reflecting the WHO definition of low, moderate and high risk transmission. It also maintains the WHO recommendations for surveillance in such areas meaning that no active surveillance is occurring in low transmission areas, whilst moderate and high transmission areas experience active screening campaigns with an average coverage rate of 80% of the 57 million people reported to be at risk[2]. Again, according to recommendations from the WHO, surveillance in moderate risk areas was simulated bi-annually and high transmission areas done annually.

As the model represents three hypothetical risk transmission areas based on low, moderate and high prevalence, the annual percentage reduction of cases per year was extracted from the model respective to the three risks areas under evaluation. The estimated number of cases was then forecasted into the future using the 6228 *T.b. gambiense* cases reported in 2013 across 13 endemic nations as a baseline. Estimations from the model and WHO reported cases up to 2014 were compared for validation of the model's predictive capacity. Forecasting estimates and current estimates for reported cases is depicted in *Figure 1*. When the mean of control strategies (with and without vector control) from 2012 to 2014 was compared to the data currently available, the model predicted cases well (R^2= 0.8301), however the available data is limited and the model's predictive ability will need to be assessed continually. Although control will reduce the number of cases over time, these reductions are expected to plateau over the next decade leading to delays in meeting elimination targets. However, if elimination strategies that include increased surveillance in low transmission areas, innovative technologies to improve coverage or targets to interrupt vector transmission (Elimination programs I, II and III) are implemented; the outcome would result in achieving near 2000 cases or less across Africa by 2020. Optimal declines were observed in Elimination II and III, where less than 500 cases could be feasible by 2020, and also contain strategies that have the highest probability of elimination[1].

Figure 1. Number of cases (households) actual (WHO surveillance) and forecasted[1,3] till 2020

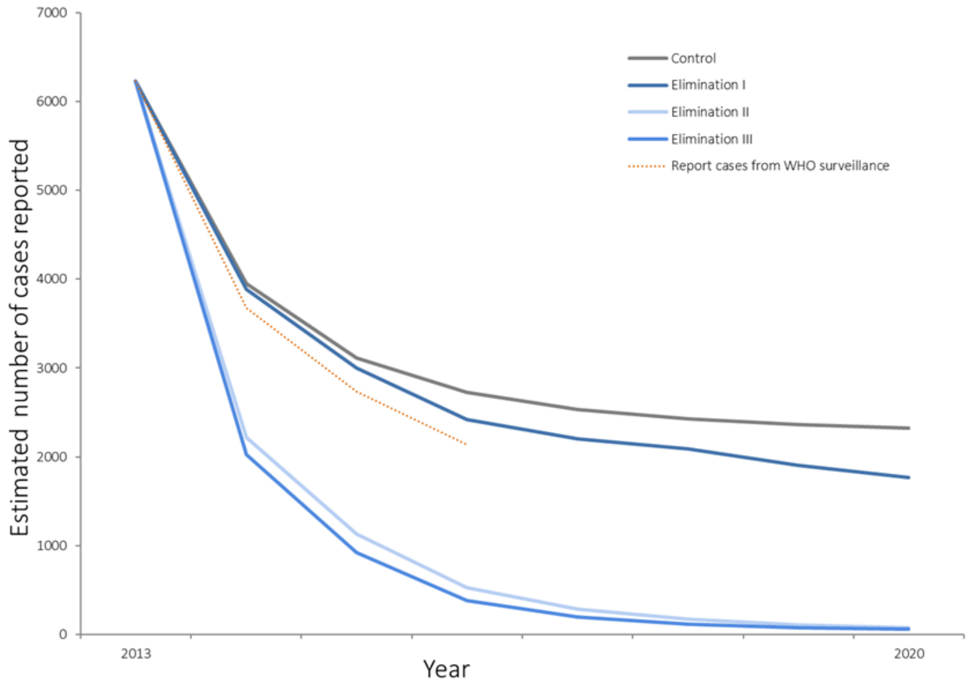

Table 5. Number of cases (households) forecasted (Stone & Chitnis 2015, Sutherland et al 2017) till 2020 for all programs: Control, Elimination I, Elimination II, and Elimination III (based on 500 simulations)

Year	Mean	SD	SE	95% CI low	95% CI high	Min	Max
**Baseline 2013**	**6228**	**0**	**0**			**6228**	**6228**
				**Control**			
**2014**	3951	261	12	3950	4297	3343	5252
**2015**	3109	365	16	3108	3381	2593	5086
**2016**	2725	404	18	2724	2964	2245	5030
**2017**	2532	420	19	2530	2753	2079	5002
**2018**	2425	428	19	2423	2637	1983	4987
**2019**	2361	432	19	2359	2568	1916	4978
**2020**	2319	435	19	2318	2523	1863	4973
				**Elimination I**			
**2014**	3889	193	9	3888	4230	3343	4526
**2015**	3000	202	9	2999	3263	2593	4228
**2016**	2421	144	6	2420	2633	1997	3709
**2017**	2201	137	6	2200	2394	1745	3633
**2018**	2092	148	7	2092	2276	1582	3602
**2019**	1902	224	10	1901	2068	979	3495
**2020**	1768	283	13	1766	1922	701	3465
				**Elimination II**			
**2014**	2217	633	28	2215	2412	943	3395
**2015**	1133	421	19	1131	1232	326	2114
**2016**	523	239	11	522	569	108	1333
**2017**	281	156	7	280	306	42	1158
**2018**	167	114	5	166	182	17	1066
**2019**	108	89	4	108	117	6	961
**2020**	76	74	3	76	82	2	892
				**Elimination III**			
**2014**	2023	639	29	2020	2200	724	3177
**2015**	917	420	19	915	997	133	1834
**2016**	383	229	10	383	417	20	1015
**2017**	197	142	6	197	215	3	881
**2018**	117	99	4	117	128	1	817

**2019**	77	75	3	77	84	0	743
**2020**	56	61	3	55	61	0	695

A.3.2 Out-of-pocket (OOP) Household health expenditures related to T.b. gambiense

A cost function for per household OOP expenditure was then developed taking into consideration that a family member or friend would attend the treatment clinic with the diagnosed individual, and was calculated according to Equation 3 in the main manuscript. The average cost of treatment for stage 1 and 2 is used as the final mean OOP costs per program (refer to *Table 6*).

Table 6. Costs and resource use related to OOP household expenditures

Short name	OOP Description	Cost	Source
One time OOP			
c_{fee}	Hospital entry fees (one time)	$2.52	Matemba, 2010
$C_{transportation}$	Transportation (roundtrip)	$9.65	Matemba, 2010
Per diem OOP			
$C_{accomodation}$	Accommodation per diem	$1.90	Matemba, 2010
c_{meals}	Meals per diem	$2.28	Matemba, 2010
tx_{days}	Days of treatment		
	Pentamidine	12	Steinmann et al
	NECT	14	Steinmann et al
	fexinidazole	10	Steinmann et al
	oxaborole	1	Steinmann et al
r_{days}	Days of recovery (related to treatment)		
	pentamidine	7	Assumption
	NECT	7	Assumption
	fexinidazole	4	Assumption
	oxaborole	2	Assumption

HAT *T.b. gambiense* affects rural populations across Sub-Saharan Africa, hence mean consumption (C) was based on the gross national income (GNI) of the endemic nations. Non-medical expenses (NM) were estimated using food expenditures as a proxy and it was assumed on average that 66.3% of a rural household income (~ $320 annual) was spent on food expenditures [5].

Table 7. Summary of inputs for the Financial Protection Analysis (FPA)

Description									95% Confidence (CI)		Source
**Households (cases)**	**Mean**	**Distribution**	**alpha**	**beta**	**SD**	**SE**	**MIN**	**MAX**	**low**	**high**	
Control **2013**	6228										Franco et al, 2017
Control 2020	2333	Normal			435	19	1863	4973	2318	2523	Stone & Chitnis, France et al 2017
**Elimination I** 2020	1774	Normal			283	13	701	3465	1766	1922	HAT model (Stone & Chitnis), projections
**Elimination II** 2020	77	Normal			74	3	2	892	76	82	HAT model (Stone & Chitnis), projections
**Elimination III** 2020	57	Normal			61	3	0	695	55	61	HAT model (Stone & Chitnis), projections
**Income (C), annual**	**Median**										
**Income - GNI**	$1,360	Gamma	2	835	3843	1066	330	12640	2438	3596	World Bank 2013
**Income - lower income GNI**	$575	Gamma	25	23	283	115	330	980	538	722	World Bank 2013
**Income - lower-middle income GNI**	$2,085	Gamma	25	83	833	417	1360	2970	1717	2533	World Bank 2013
**Income - middle-upper income GNI**	$9,450	Gamma	17	541	3916	2261	4850	12640	6421	11539	World Bank 2013
**Non-medical expenses (NM), annual**											
**Non-medical expenses (i.e food)**	$320	Gamma	319.566	1							Lutumba 2007, Chauvin 2012
**OOPs, Medical expenses related to HAT *Tbg* (M)**											
	**Mean**										
Control 2013	$151	Gamma	151.02	1	9.136						Matemba 2010
Control 2020	$151	Gamma	151.02	1	9.136						Matemba 2010
Elimination I 2020	$3	Gamma	2.52	1							Matemba 2010
Elimination II 2020	$3	Gamma	2.52	1							Matemba 2010,
Elimination III 2020	$3	Gamma	2.52	1							Matemba 2010
**Other inputs**											
CHE threshold	10% and 25%										World Bank 2017
**Poverty line**	1.9										World Bank 2017
**Discount rate**	3%										WHO-CHOICE

C=consumption, income; GNI=gross national income, NM=non-medical expense, M=medical expense, OOP=out-of-pocket payments, CHE = catastrophic health expenditure

Table 8. Sensitivity and scenario analyses results for FPA, sub-Saharan Africa

	Control	Control	Elimination I	Elimination II	Elimination III
Total cases (N)	6228*	2319	1768	76	56
PROPORTION **of households that pay Medical expenses related to HAT *Tbg*,** 75%					
Impoverishing	21.97%	21.99%	15.64%	15.72%	15.44%
Immiserizing	30.97%	30.94%	30.99%	30.78%	30.97%
Catastrophic (CHE) at 10%	62.88%	62.86%	1.26%	1.26%	1.27%
Difference in % from Control 2013	NA	-0.02%	-61.62%	-61.62%	-61.61%
Catastrophic (CHE) at 25%	30.97%	30.94%	0.50%	0.50%	0.47%
Difference in % from Control 2013	NA	-0.03%	-30.47%	-30.47%	-30.50%
PROPORTION **of households that pay Medical expenses related to HAT *Tbg*, 60%**					
Impoverishing	21.97%	21.97%	15.56%	15.45%	15.80%
Immiserizing	30.97%	31.03%	31.00%	31.01%	30.56%
Catastrophic (CHE) at 10%	62.90%	62.89%	1.28%	1.27%	1.23%
Difference in % from Control 2013	NA	-0.01%	-61.62%	-61.63%	-61.67%
Catastrophic (CHE) at 25%	30.97%	30.99%	0.51%	0.51%	0.45%
Difference in % from Control 2013	NA	0.02%	-30.46%	-30.46%	-30.52%
**PROPORTION of households that pay Medical expenses related to HAT *Tbg*, 36%**					
Impoverishing	17.85%	17.88%	15.54%	15.51%	15.56%
Immiserizing	30.96%	30.97%	30.94%	30.91%	30.96%
Catastrophic (CHE) at 10%	22.67%	22.68%	0.45%	0.46%	0.45%
Difference in % from Control 2013	NA	0.01%	-22.21%	-22.21%	-22.22%
Catastrophic (CHE) at 25%	11.15%	11.18%	0.18%	0.18%	0.15%
Difference in % from Control 2013	NA	0.03%	-10.97%	-10.97%	-11.00%
POVERTY LINE= USD 1.25 per [illegible]					
Impoverishing	24.08%	24.08%	16.89%	16.59%	17.00%
Immiserizing	18.43%	18.45%	18.38%	18.50%	18.30%
Catastrophic (CHE) at 10%	62.89%	62.95%	1.27%	1.31%	1.21%

Difference in % from Control 2013	NA	0.06%	-61.62%	-61.58%	-61.68%
**Catastrophic (CHE) at 25%**	30.97%	30.96%	0.50%	0.53%	0.48%
Difference in % from Control 2013	NA	-0.01%	-30.47%	-30.45%	-30.50%
**POVERTY LINE= USD 3.1 per diem**					
**Impoverishing**	16.70%	16.68%	12.02%	12.10%	12.20%
**Immiserizing**	51.61%	51.61%	51.60%	51.29%	51.38%
**Catastrophic (CHE) at 10%**	62.89%	62.87%	1.26%	1.31%	1.20%
Difference in % from Control 2013	NA	-0.02%	-61.63%	-61.59%	-61.69%
**Catastrophic (CHE) at 25%**	30.96%	30.89%	0.51%	0.53%	0.54%
Difference in % from Control 2013	NA	-0.06%	-30.45%	-30.43%	-30.41%
**DISCOUNTING 0%**					
**Impoverishing**	22.00%	21.96%	15.67%	15.65%	15.75%
**Immiserizing**	30.95%	30.93%	30.91%	31.14%	30.93%
**Catastrophic (CHE) at 10%**	62.89%	62.83%	1.28%	1.26%	1.27%
Difference in % from Control 2013	NA	-0.06%	-61.61%	-61.63%	-61.62%
**Catastrophic (CHE) at 25%**	30.95%	30.92%	0.51%	0.54%	0.50%
Difference in % from Control 2013	NA	-0.03%	-30.44%	-30.41%	-30.44%
**DICOUNTING 6%**					
**Impoverishing**	22.00%	21.99%	15.62%	15.46%	15.44%
**Immiserizing**	30.95%	30.93%	30.92%	31.06%	30.91%
**Catastrophic (CHE) at 10%**	62.89%	62.82%	1.26%	1.24%	1.29%
Difference in % from Control 2013	NA	-0.07%	-61.62%	-61.65%	-61.60%
**Catastrophic (CHE) at 25%**	30.95%	30.93%	0.49%	0.50%	0.52%
Difference in % from Control 2013	NA	-0.02%	-30.45%	-30.44%	-30.43%

References

1 Sutherland CS, Stone CM, Steinmann P, *et al.* Seeing beyond 2020: an economic evaluation of contemporary and emerging strategies for elimination of Trypanosoma brucei gambiense. *Lancet Glob Heal* 2017;**5**:e69–79. doi:10.1016/S2214-109X(16)30237-6

2 Franco JR, Cecchi G, Priotto G, *et al.* Monitoring the elimination of human African trypanosomiasis: Update to 2014. *PLoS Negl Trop Dis* 2017;**11**:e0005585. doi:10.1371/journal.pntd.0005585

3 Stone CM, Chitnis N. Implications of Heterogeneous Biting Exposure and Animal Hosts on Trypanosomiasis brucei gambiense Transmission and Control. *PLOS Comput Biol* 2015;**11**:e1004514. doi:10.1371/journal.pcbi.1004514

4 World Health Organization (WHO). WHO Technical Report Series 984: Control and surveillance of human African trypanosomiasis. WHO. 2013.http://apps.who.int/iris/bitstream/10665/95732/1/9789241209847_eng.pdf

5 Depetris Chauvin N, Mulangu F, Porto GG. Food Production and Consumption Trends in Sub-Saharan Africa: Prospects for the Transformation of the Agricultural Sector. United Nations Development Programme, Regional Bureau for Africa 2012. http://econpapers.repec.org/RePEc:rac:wpaper:2012-011 (accessed 8 Apr 2016).
